# Structural Characterization and Evaluation of the Antioxidant Activity of Phenolic Compounds from *Astragalus taipaishanensis* and Their Structure-Activity Relationship

**DOI:** 10.1038/srep13914

**Published:** 2015-09-09

**Authors:** Wenjun Pu, Dongmei Wang, Dan Zhou

**Affiliations:** 1 College of Forestry, Northwest A & F University, Yangling, Shaanxi 712100, China

## Abstract

Eight phenolic compounds were isolated using bio-guided isolation and purified from the roots of *Astragalus taipaishanensis* Y. C. Ho et S. B. Ho (*A. taipaishanensis*) for the first time. Their structures were elucidated by ESI-MS, HR-ESI-MS, ^1^D-NMR and ^2^D-NMR as 7,2′-dihydroxy-3′,4′-dimethoxy isoflavan (**1**), formononetin (**2**), isoliquiritigenin (**3**), quercetin (**4**), kaempferol (**5**), ononin (**6**), p-hydroxybenzoic acid (**7**) and vanillic acid (**8**). Six flavonoids (compounds **1-6)** exhibited stronger antioxidant activities (determined by DPPH, ABTS, FRAP and lipid peroxidation inhibition assays) than those of BHA and TBHQ and also demonstrated noticeable protective effects (particularly quercetin and kaempferol) on *Escherichia coli* under oxidative stress. Additionally, the chemical constituents compared with those of *Astragalus membranaceus* and the structure-activity relationship of the isolated compounds were both analyzed. The results clearly demonstrated that *A. taipaishanensis* has the potential to be selected as an alternative medicinal and food plant that can be utilized in health food products, functional tea and pharmaceutical products.

Medicinal food plants are widely utilized in dietary supplements as a source of bioactive compounds that have food additive properties^1^. Research has demonstrated that bioactive compounds extracted from plants are effective in food systems and can be used as antimicrobial and antioxidant additives in the food industry^1^,^2^. Given the increasing application and use of these bioactive compounds in food, cosmetic and pharmaceutical products as replacements for synthetic antioxidants, which are often constricted due to known carcinogenic effects^3^, there is an increased interest and emphasis on the discovery and utilization of traditional medicinal food plants.

The *Astragalus* spp. has a history of being widely used in traditional medicine, coffee, tea substitutes, food and cosmetics worldwide, particularly in places such as Europe, the Middle East and Asia^4^,^5^. Nearly 2000 species have been identified and distributed around largely subtropical and temperate regions with the exception of Oceania^6^,^7^. *A. membranaceus* Bunge, which is also known as Huangqi in Chinese and *Radix Astragali* in Latin, belongs to the Leguminosae family. It was first recorded in Shennong Bencao Jing and its dry roots have been used in traditional Chinese medicine since 1800^8^,^9^,^10^,^11^. Currently, the products of *Radix Astragali*, such as Astragali tea and over-the-counter dietary supplement capsules, are available in U.S. health food markets^12^,^13^. In China, Astragalus-jujube-wolfberry tea is a well-known anti-cancer tea remedy that can enhance body vitality and immunity. The study of its chemical constituents demonstrated that more than 100 components of *Radix Astragali* have been identified and the main constituents are flavonoids, polysaccharides, saponins and amino acids^14^,^15^. Many pharmacology studies have revealed that the flavonoids of *Radix Astragali* exhibit noticeable antioxidant activity^16^,^17^ and pharmacological activities such as immunoregulatory effects^18^, a decrease in the damage caused by hypoxia^19^ and amelioration of chronic fatigue syndrome^20^.

*A. taipaishanensis* is a species native to China that belongs to the *Astragalus* genus, which is only distributed in the Taibai Mountain region. In the Taibai Mountains, teas, decoctions and infusions are prepared from the roots of *A. taipaishanensis* as folk medicine and functional tea to enhance immunity and to improve stamina and strength^21^; however, until now, there have been no reports on the phytochemicals or bioactive compounds of this plant. In the following study, the bioactive components present in the roots of *A. taipaishanensis* were isolated and extracted to discover how to better utilize this plant and also attempt to gain a deeper understanding of the correlation between the health benefits of the compounds and reduced risk of diseases. The antioxidant activities of these isolated compounds were tested using several *in vitro* assays (DPPH, ABTS, FRAP and lipid peroxidation inhibition assays) along with an additional test to assess their protective effects on *Escherichia coli* subjected to oxidative stress by hydrogen peroxide. The results indicated that the roots of *A. taipaishanensis* had similar constituents and antioxidant capacities to those of *A. membranaceus*, indicating that it may have the potential to be developed for use in supplementary medicine and pharmaceutical products and functional food ingredients.

## Results

### TPC, TFC and antioxidant activities of extracts and isolated fractions

The TPC and TFC of the ethanolic extracts (EE) and 4 isolated fractions (PEF, EAF, BF, and WF) of *A. taipaishanensis* were analyzed (Table 1). The results demonstrated that the EAF had the highest content of TPC (46.41 ± 0.34 mmol equiv. GAE/100 g) and TFC (185.45 ± 1.04 mmol equiv. QUE/100 g). To identify the fraction with the greatest bioactivity, four *in vitro* antioxidant activities were observed in each fraction and the most bioactive fraction was then selected for further isolation and purification. The results are displayed in Table 1. For the ABTS^•+^ and FRAP assay, the EAF had the highest ABTS scavenging activity and reducing ability with values of 994.50 ± 4.21 μmol trolox/g and 685.67 ± 3.21 μmol trolox/g, respectively. For the DPPH and lipid peroxidation assays, the IC_50_ values of DPPH and lipid peroxidation ranged from 0.059 ± 0.002 mg/mL (EAF) to 0.99 ± 0.004 mg/mL (WF) and 1.95 ± 0.009 mg/mL (EAF) to 6.48 ± 0.039 mg/mL (WF), respectively. In conclusion, the EAF was determined to be the fraction with the greatest antioxidant activity; therefore, later isolation and purification efforts were focused on the EAF.

### Structure elucidation of isolated compounds

**Figure 1:**
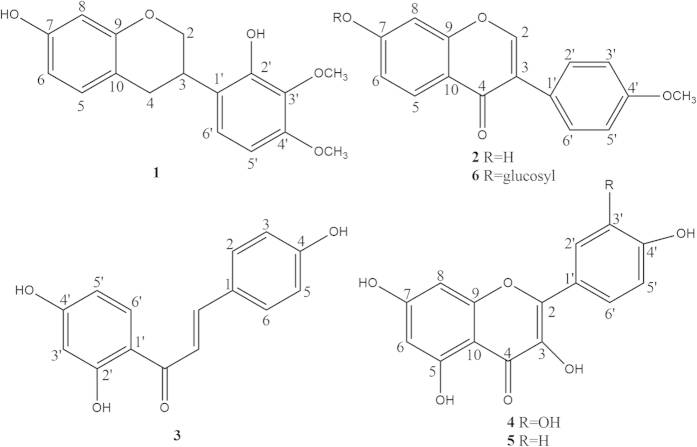
Structures of compounds 1–6.

**Figure 2:**
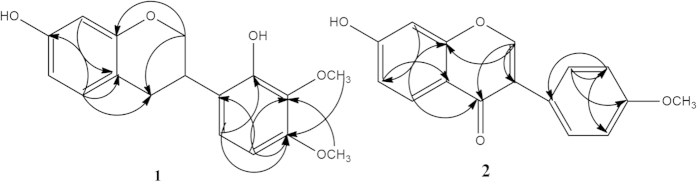
Key HMBC correlations of compounds 1 and 2.

Eight compounds were isolated and identified from the roots of *A. taipaishanensis*. The NMR data of the compounds are shown in Tables 2 and 3. The structures of compounds **1-8** and the key HMBC correlation of compounds **1** and **2** are illustrated in Figs 1 and 2.

Compound **1** was isolated as white needle-shaped crystals. Its molecular formula was determined to be C_17_H_18_O_5_ on the basis of HR-ESI-MS: *m*/*z* 301.1229 [M-H]^−^ and ESI-MS: *m/z* 301.15 [M-H]^−^. The ^13^C-NMR and DEPT spectra exhibited signals for two OCH_3_, two CH_2_ (all aliphatic), six CH (five aromatic, one aliphatic) and seven quaternary carbons (four O-bearing and three aromatic), respectively. The ^1^H-NMR spectrum of compound **1** displayed two -OCH_3_ signals at *δ* 3.78 (3H,s) and *δ* 3.79 (3H,s), five aromatic proton signals at *δ* 6.22 (1H,s), *δ* 6.30 (1H,d,*J* = 8.0 Hz), *δ* 6.44 (1H,d,*J* = 8.4 Hz), *δ* 6.75 (1H,d,*J* = 8.8 Hz) and *δ* 6.85 (1H,d,*J* = 8.4 Hz), which represented the structure containing the AMX spin-coupling. When compared with published literature data, compound **1** was identified as 7, 2′-dihydroxy-3′, 4′-dimethoxy-isoflavane^22^.

Compound **2** was obtained as white needle crystal. Its molecular formula was established as C_16_H_12_O_4_ on the basis of HR-ESI-MS: m/z 267.0930 [M-H]^−^ and ESI-MS: *m/z* 267.17 [M-H]^−^. ^13^C-NMR and DEPT spectrum showed signals for one OCH_3_, eight CH (seven aromatic, one alkene) and seven quaternary carbons (one carbonyl, two O-bearing and four aliphatic), respectively. ^1^H-NMR spectrum of compound 2 exhibited one -OCH_3_ signal at *δ* 3.80(3H, s) and three aromatic proton signals at *δ* 6.88(1H, d, *J* = 2.5 Hz), *δ* 6.95(1H, dd, *J* = 2.0, 2.0 Hz), *δ* 7.98 (1H, d, *J* = 8.5 Hz), respectively. The signal at *δ* 8.34 (1H, s) was an obvious signal of isoflavone at the H-2 position. The AA′BB′ spin-coupling system, with the signals at *δ* 7.00 (2H, d, *J* = 9.0 Hz), *δ* 7.52 (2H, d, *J* = 8.5 Hz), was ascribed to the four protons of the B-ring. After being compared with published literature values, compound **2** was identified as formononetin^23^.

Compound **6** was isolated as a white amorphous powder. ESI-MS: *m/z* 431.32 [M+H]^+^. Its molecular formula was determined to be C_22_H_22_O_9_ based on the ESI-MS and NMR. Compared with compound **2**, it had the same isoflavone skeleton. Additionally, the mass spectra and NMR spectra showed signals corresponding to a single sugar unit. The ^1^H-NMR spectrum of compound **6** displayed strong signals indicating anomeric protons at *δ* 5.16 (1H, d, *J* = 4.8 Hz). The sugar unit was examined by TLC analysis. Acid hydrolysis of compound 6 resulted in a product of D-glucose, which was identified by a comparison with an authentic sample. Based on the coupling constant *J* = 4.8 Hz greater than 4.0 Hz, the sugar configurations were identified as β-D-glucose. In addition, there was a remarkable upfield shift (Δ*δ* 1.14) for the C-7 of compound **6** (161.91) compared to compound **2** (163.05), indicating that the sugar unit was attached to C-7. After comparison with published literature data, compound **6** was identified as ononin^24^.

Compound **3** was isolated as a yellow amorphous powder. ESI-MS: *m/z* 255.49 [M-H]^−^. The ^13^C-NMR and DEPT spectra exhibited signals for nine CH (seven aromatic, two alkene) and six quaternary carbons (one carbonyl, three O-bearing and two aliphatic), respectively. Its molecular formula was determined to be C_15_H_12_O_4_ based on the results from ESI-MS and NMR. The ^1^H-NMR spectrum of compound **3** exhibited three aromatic proton signals at *δ* 6.30 (1H, d, *J* = 2.4 Hz), *δ* 6.43 (1H, dd, *J* = 2.4, 2.4 Hz), and *δ* 7.99 (1H, d, *J* = 8.8 Hz), and it was ascribed to three protons of the A-ring of flavone. The signal at *δ* 7.63 (1H, d, *J *= 5.2 Hz) and *δ* 7.81(1H, d, *J *= 15.2 Hz) were obvious signals of conjugate protons; moreover, there was an AA′BB′ spin-coupling system with signals at *δ* 6.87 (2H, d, *J* = 8.8 Hz), *δ* 7.65(2H, d, *J *= 1.2 Hz), which was attributed to the four protons of the B-ring. When compared with published literature data, compound **3** was identified as isoliquiritigenin^25^.

Compound **4** was isolated as a yellow amorous powder. ESI-MS: *m/z* 301.29 [M-H]^−^. The ^13^C-NMR and DEPT spectra exhibited signals for five CH (all aromatic) and ten quaternary carbons (one carbonyl, five O-bearing, four aliphatic). Its molecular formula was determined to be C_15_H_10_O_6_ based on the results of ESI-MS and NMR. In the ^1^H-NMR spectrum, an ABX spin-coupling system was ascribed to the three protons of the B-ring of the flavone, with signals at *δ* 6.89 (1H, d, *J* = 6.8 Hz), *δ* 7.54 (1H, dd, *J* = 2.0 Hz, *J* = 2.0 Hz), and *δ* 7.65 (1H, d, *J* = 8.5 Hz), and with respect to published literature data, compound **4** was identified as quercetin^26^.

Compound **5** was isolated as a yellow amorous powder. ESI-MS: *m/z* 284.96 [M-H]^−^. The ^13^C-NMR and DEPT spectra displayed signals for six CH (all aromatic) and nine quaternary carbons (one carbonyl, four O-bearing, four aliphatic). Its molecular formula was determined to be C_15_H_10_O_5_ based on the ESI-MS and NMR spectra. Compound **5** was found to have the same skeleton as compound **4**. In the ^1^H-NMR spectrum, the AA′BB′ spin-coupling system with signals at *δ* 6.92 (2H, d, *J* = 8.5 Hz) and *δ* 8.04 (1H, d, *J* = 8.5 Hz) was ascribed to the four protons of the B-ring. The signals at *δ* 6.19 (1H, d, *J* = 2.0 Hz) and *δ* 6.45 (1H, d, J = 2.0 Hz) were meta-substituted protons of the A-ring. When compared with published literature data, compound **5** was identified as kaempferol^26^.

Compound **7** and compound **8** were identified by TLC analysis and a comparison with an authentic sample. Compound **7** was identified as p-hydroxy benzoic acid, and compound **8** was identified as vanillic acid^27^.

To the best of our knowledge, this is the first report of the characterization of phenolic compounds **1–8** isolated from *A. taipaishanensis*; furthermore, the ^2^D-NMR data of compounds **1** and **2** were presented for the first time in this study.

### *In vitro* antioxidant assays of isolated compounds

Phenolic compounds possess biologically beneficial activities and are frequently utilized as antioxidant, antiallergic and antidiabetic reagents^28^. In our study, the compounds **1–6** were tested for antioxidant by using four *in vitro* antioxidant assays and protective effect on *E. coli* under peroxide stress. The results showed in the Table 4. For the ABTS assay, all the tested compounds showed strong antioxidant capacity compared to BHA and TBHQ. In particular, compounds **4** and **5** had comparative higher antioxidant capacity with ABTS values over 30 mmol trolox/g. FRAP assay, compounds **4, 5** and **1** had higher antioxidant activity compared to BHA (5.83 ± 0.08 mmol trolox/g) and TBHQ (6.57 ± 0.13 mmol trolox/g), and the values were 14.18 ± 0.13, 14.13 ± 0.16 and 10.77 ± 0.11 mmol trolox/g, respectively. The results of DPPH assay demonstrated that all the tested compounds had stronger DPPH radical-scavenging ability than the standards (BHA and TBHQ). Compounds **4, 5, 1** and **3** in particular had relatively higher antioxidant activity, in which the IC_50_ values were lower than 1 μM. The lipid peroxidation assay was carried out using egg yolk as a lipid-rich medium. For this assay, all the tested compounds exhibited a particular capacity for lipid peroxidation inhibition. In particular, compounds **4, 5** and **1** had comparatively higher lipid peroxidation potential with IC_50_ values 1.65 ± 0.08, 1.30 ± 0.09 and 1.87 ± 0.07 μM, respectively, all of which were lower than 2 μM.

### Protective effect on *E. coli* under peroxide stress

**Figure 3:**
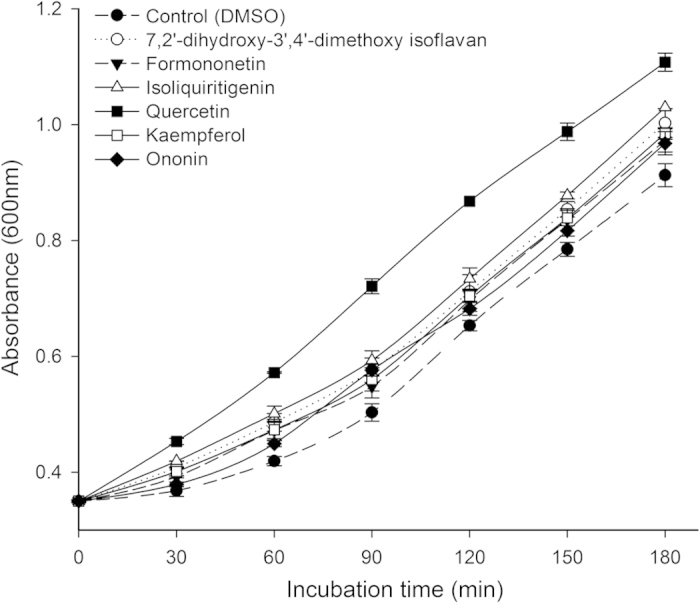
Influence of pretreatment with different isolated compounds on the growth of *E. coli* under H_2_O_2_ peroxide stress. **Note:** mean ± SD, N = 3.

**Figure 4:**
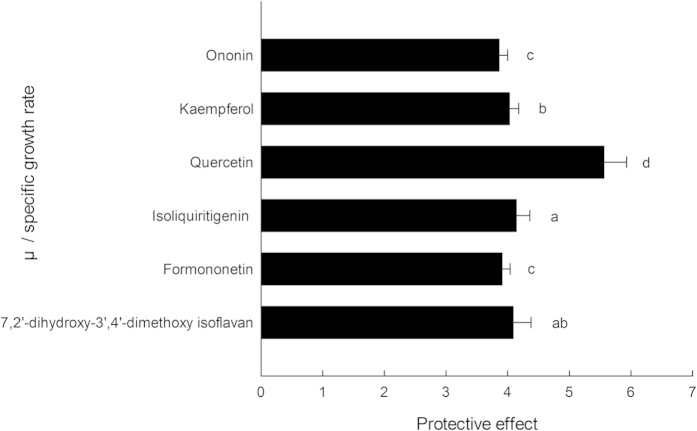
The relative protective effect of isolated compounds on *E. coli* under peroxide stress. **Note:** The specific growth rate of *E. coli* in the medium containing the compounds and 6.5 mM H_2_O_2_/the specific growth rate in medium containing only 6.5 mM H_2_O_2_ (mean ± SD, N = 3), t = 30 min.

Six of the isolated compounds were tested on *E. coli* growth under peroxide stress. We evaluated and compared the specific growth rate (μ) of *E. coli* cells by pretreating them with the 6 compounds before subjecting them to oxidative stress damage caused by H_2_O_2_ for 30 min. The absorbance (OD_600_) and μ values are displayed in Figs 3 and 4, respectively. The higher values of the μ and the OD_600_ were indicative of a higher antioxidant potential for the compounds. The results demonstrated that all the compounds had the ability to increase the cell growth rate under oxidative stress by 3.86- to 5.56-fold compared to untreated cells subjected to oxidative stress for 30 min. The compound with the highest activity was compound **4**, which resulted in a growth rate of 5.56, followed by compound **3** and compound **1**, which resulted in growth rates of 4.14 and 4.09, respectively.

## Discussion

*A. membranaceus* and *A. membranaceus* var. *mongholicus* are mainly grown in North, Northeast and Northwest China and have been widely used for 2000 years^29^. Flavonoids, triterpene saponins and polysaccharides are the main constituents of this plant. Currently, almost 30 flavonoids have been isolated from *A. membranaceus*^30^,^31^, most of them being isoflavones, isoflavans and flavonols. Isoflavones in particular are major active ingredients in *A. membranaceus*^32^ and were selected as marker compounds to evaluate the quality of *A. membranaceus*. Due to its known multiple health benefits, the natural sources of *A. membranaceus* are diminishing yearly^33^. In an attempt to address this shortage by exploring alternative plant options, we carried out a study using bio-guided isolation to isolate 8 phenolic compounds from the roots of *A*. *taipaishanensis* for the first time, examining and evaluating their bioactivities by *in vitro* radical scavenging, ferric reducing power and lipid peroxidation in addition to assessing their protective ability on *E. coli* cells subjected to oxidative stress damage caused by H_2_O_2_.

We isolated 7, 2′-dihydroxy-3′,4′-dimethoxy isoflavan (**1**), formononetin (**2**), isoliquiritigenin (**3**), quercetin (**4**), kaempferol (**5**), ononin (**6**), p-hydroxy benzoic acid (**7**) and vanillic acid (**8**) from the roots of *A*. *taipaishanensis*. When compared with published literature, it was discovered that these 8 compounds isolated from *A*. *taipaishanensis* were all present in *A. membranaceus*, particularly isoliquiritigenin, formononetin, ononin, kaempferol, quercetin, which are the main beneficial flavonoids in *A. membranaceus*; therefore, it is possible to infer that *A*. *taipaishanensis* would contain similar flavonoid constituents to those of *A. membranaceus.* This could also explain the popular use the roots of *A. taipaishanensis* as folk medicine and functional tea to enhance the immune system and improve stamina and strength.

By comparing the results of four *in vitro* antioxidant assays, it was evident that quercetin and kaempferol demonstrated comparatively higher levels of antioxidant activity in the assays, whereas the formononetin and ononin had the lowest antioxidant potential, and the 7, 2′-dihydroxy-3′,4′-dimethoxy isoflavan and isoliquiritigenin had moderate antioxidant capacity. Compared with BHA and TBHQ, all the tested compounds demonstrated higher radical scavenging abilities against the ABTS and DPPH assays, whereas for the lipid peroxidation assay, all the compounds had lower activity than BHA and TBHQ. This result demonstrated that all the compounds isolated from the *A. taipaishanensis* had good radical scavenging activity. In particular, the results of the antioxidant assays indicated that all the tested compounds had certain antioxidant properties although their activity levels were significantly different. Among the 6 compounds that were tested, we found that the compounds with a flavonol skeleton, such as quercetin (**4**) and kaempferol (**5**), always had the highest antioxidant activities. In contrast, 7, 2′-dihydroxy-3′,4′-dimethoxy isoflavan (**1**) had a slightly lower antioxidant activity than flavonol followed by chalcone (isoliquiritigenin, **3**) and the isoflavone (formononetin, **2**), and isoflavone glucoside (ononin, **6**) had the lowest antioxidant capacities. These results were consistent with previous reports stating that the number and position of hydroxyl groups and the different flavonoid skeletons would largely influence the radical scavenging ability. Previous reports stated that the 3-OH group would significantly increase the radical scavenging power^34^,^35^, and among all the tested compounds, only quercetin and kaempferol had the 3-OH group, both of which were found to have the highest anti-radical activities. In contrast, the formononetin and ononin, which had no 3-OH group, had the lowest radical scavenging power^36^; moreover, the presence of ortho-dihydroxy groups on the B-ring or A-ring could have also enhanced the radical scavenging potential, which is a possible explanation as to why quercetin had slightly higher antioxidant activity in comparison to kaempferol. In addition, the presence of an *α, β*-double bond in isoliquiritigenin made its anti-radical potential evident^36^,^37^.

In conclusion, the present study, to the best of our knowledge, was the first time a comprehensive study was carried out on the phytochemicals and antioxidant capacity of the *A. taipaishanensis.* We isolated 6 flavonoid compounds and 2 phenolic compounds from *A. taipaishanensis*, all of which were isolated for the first time from this species. Additionally, we found that the main chemical components and the bioactivity of *A. taipaishanensis* were similar to those found in *A. membranaceus*, which is recorded in the pharmacopoeia as a medicinal and food plant. The antioxidant activities and structure-activity relationships of these isolated compounds were also evaluated, and the results revealed that all the tested compounds exhibited noticeable antioxidant activity compared to BHA and TBHQ. These findings demonstrate the potential of *A. taipaishanensis* to serve as a valuable natural resource in the medicinal and food industry and its ability to be used as a possible alternative in response to the diminishing supply of *A. membranaceus.*

## Methods

### General

ESI-MS was performed on an Agilent 1200 HPLC with the 6130 SQD system and a Waters XBridge C_18_ column (50 × 4.6 mm, 3.5 μm, Waters). HR-ESI-MS was performed on 1260 HPLC combined with 6250 ESI-Q-TOF MS (Agilent, USA). HPLC separation was performed on an Agilent 1260 HPLC with a Zobax Eclipse Plus C_18_ column (4.6 × 250 mm, 5 μm, Agilent). ^1^H-and ^13^C-spectra were measured with a Bruker AVANCE III spectrometer (500 MHz). Thin layer silica gel and column gel were both purchased from Qingdao Ocean Company (China). Sephadex LH-20 was purchased from GE Healthcare Bioscience AB (Sweden).

### Chemicals and reagents

The following chemicals and reagents were used: 1,1-diphenyl-2-picrylhydrazyl (DPPH), 2,4,6-tripyridyl-s-triazine (TPTZ), 2,2-azino-bis(3-ethyl-benzothiazoline-6-sulphonic acid) di-ammonium salt (ABTS), 6-hydroxy-2,5,7,8-tetramethylchroman-2-carboxylic acid (Trolox), (Sigma-Aldrich Co., St. Louis, USA); thiobarbituric acid (TBA) (Guangdong Guanghua Chemical Factory Co., Ltd. PR China); sodium borohydride (NaBH4), vanillin, aluminum chloride, acetic acid, hydrochloric acid, sodium dihydrogen phosphate, disodium hydrogen phosphate, sodium chloride, potassium chloride, potassium dihydrogen phosphate, sodium carbonate, sodium acetate, ferric trichloride hexahydrate (FeCl_3_·6H_2_O), potassium persulfate (Tianjin Bodi Chemical Co., Ltd, PR China); tetrahydrofuran (THF) (Shenzhen Guanghua Technology Co., Ltd, PR China); quercetin, formononetin, glucose (Shanghai Source Leaf Biological Technology Co., Ltd. PR China); yeast extract, tryptone (Oxoid Ltd., Basingstoke, Hampshire, England). All the chemicals used including the solvents were of analytical grade.

### Plant material

Five-year-old roots of *A. taipaishanensis* were collected on July 2013 at the Taibai Mountains of Shaanxi province, China. The specimen was kept in the herbarium of Northwest A&F University, Yangling, China.

### Extraction and isolation

The dried roots of *A. taipaishanensis* (3.0 kg) were extracted five times each with 95% ethanol (10 L) for 24 h at room temperature. The ethanol extracts (EE, 488 g) were evaporated in vacuum at 45 °C and dried to solid at 35 °C. The EE (420 g) was further partitioned with petroleum ether-water, ethyl acetate-water and n-BuOH-water to obtain the 4 fractions: petroleum ether fraction (PEF, 24 g), ethyl acetate fraction (EAF, 18 g), n-butanol fraction (BF, 40 g) and water fraction (WF, 374 g), respectively. The yield of EE and the four fractions was 16.27%, 5.71%, 4.29%, 9.52% and 80.48%, respectively.

The EAF (18 g) was purified using a silica gel column (200–300 mesh, 1000 × 60 mm) and successively eluted with a petroleum ether/ethyl acetate gradient elution (90:10 to 0:100) to yield 5 fractions (Fr. 1-Fr. 5). Fr. 1 (2.2 g, 80:20) was subjected to a silica gel column (200–300 mesh, 600 × 35 mm) using a petroleum ether/ethyl acetate gradient elution (90:10 to 0:100) to yield 3 fractions (Fr. 1.1–1.3). Fr. 1.3 (1.5 g, 1:1) was subjected to a silica gel column (200–300 mesh, 600 × 20 mm) using a dichloromethane/benzene gradient elution (100:0 to 0:100) to obtain 3 fractions (Fr. 1.3.1–1.3.3). Fr. 1.3.2 (920 mg, 2:1) was purified by silica gel column (200–300 mesh, 600 × 15 mm) and eluted with a petroleum ether/ethyl acetate gradient elution (90:10 to 0:100) to obtain compound **1** (110 mg). Fr. 3 (3.5 g, 1:1) was subjected to a silica gel column (200–300 mesh, 600 × 35 mm) using a petroleum ether/acetone gradient elution (100:0 to 0:100) to yield 4 sub-fractions (Fr. 3.1–3.4). Fr. 3.2 (950 mg, 4:1) was further isolated by using a silica gel column (200–300 mesh, 600 × 15 mm) and an ethyl acetate/benzene gradient elution (100:0 to 0:100) to yield 3 fractions (Fr. 3.2.1–3.2.3). Fr. 3.2.2 (410 mg, 2:1) was further purified by HPLC (Agilent Zobax Eclipse Plus C_18_, 4.6 × 250 mm, 30% MeOH, 70% H_2_O, 0.8 mL/min, UV 254 nm) to obtain compound **2** (35 mg). Fr. 3.4 (1.9 g, 1:2) was subjected to a silica gel column (200–300 mesh, 600 × 20 mm) using a petroleum ether/acetone gradient elution (100:0 to 0:100) to yield 3 sub-fractions (Fr. 3.4.1–3.4.3). Fr. 3.4.2 (1.3 g, 2:1) was subjected to a silica gel column (200–300 mesh, 600 × 15 mm) to further isolation by eluting with dichloromethane/benzene (100:0 to 0:100) and yield 3 fractions (Fr. 3.4.2.1-3.4.2.3). Fr. 3.4.2.2 (830 mg, 2:1) was purified by using a silica gel column (200–300 mesh, 600× 15 mm) and a petroleum ether/ethyl acetate gradient elution (100:0 to 0:100) to yield 2 fractions (Fr. 3.4.2.2.1–3.4.2.2.2). Fr. 3.4.2.2.1 was further purified by HPLC (Agilent Zobax Eclipse Plus C_18_, 4.6 × 250 mm, 30% MeOH in H_2_O, 0.8 mL/min, UV 254 nm) to obtain compound **3** (25 mg). Compound **7** (22 mg) was purified by HPLC (Agilent Zobax Eclipse Plus C_18_, 4.6 × 250 mm, 30% MeOH in H_2_O, 0.8 mL/min, UV 254 nm) from Fr. 3.4.2.2.2. Compound **8** (23 mg) was purified by HPLC (Agilent Zobax Eclipse Plus C_18_, 4.6 × 250 mm, 30% MeOH, 70% H_2_O, 0.8 mL/min, UV 254 nm) from Fr. 3.4.2.3. Fr. 4 (3.9 g, 1:4) was subjected to a silica gel column (200–300 mesh, 600 × 35 mm) using a petroleum ether/acetone gradient elution (100:0 to 0:100) to yield 4 sub-fractions (Fr. 4.1–4.4). Fr. 4.2 (1.4 g, 3:1) was subjected to a silica gel column (200–300 mesh, 600 × 20 mm) using a dichloromethane/acetone gradient elution (100:0 to 0:100) to yield 3 fractions (Fr. 4.2.1–4.2.3). Fr. 4.2.2 (210 mg, 2:1) was applied to a Sephadex LH-20 column (1000 × 15 mm) and eluted with MeOH to obtain compound **4** (21 mg). Fr. 4.3 (1.2 g, 2:1) was subjected to a silica gel column (200–300 mesh, 600 × 20 mm) using a dichloromethane/acetone gradient elution (100:0 to 0:100) to obtain 2 fractions (Fr. 4.3.1–4.3.2). Fr. 4.3.1 (320 mg, 2:1) was further purified by HPLC (Agilent Zobax Eclipse Plus C_18_, 4.6 × 250 mm, 25% MeOH, 75% H_2_O, 0.8 mL/min, UV 254 nm) to obtain compound **5** (24 mg). Fr. 5 (2.3 g) was subjected to a silica gel column (200–300 mesh, 600 × 35 mm) using a petroleum ether/acetone gradient elution (100:0 to 0:100) to yield 4 fractions (Fr. 5.1–5.4). Fr. 5.4 (1.1 g, 1:2) was subjected to a silica gel column (200–300 mesh, 600 × 15 mm) using a dichloromethane/acetone gradient elution (100:0 to 0:100) to yield 2 fractions (Fr. 5.4.1–5.4.2). Fr. 5.4.2 (630 mg, 1:2) was applied to a Sephadex LH-20 column (1000 × 15 mm) and eluted with MeOH to obtain Fr. 5.4.2.1 (250 mg). Compound **6** (36 mg) was purified by HPLC (Agilent Zobax Eclipse Plus C_18_, 4.6 × 250 mm, 25% MeOH, 75% H_2_O, 0.8 mL/min, UV 254 nm) from Fr. 5.4.2.1.

### Acid hydrolysis of compound 6

Compound **6** (5.0 mg) was dissolved in 5.0 mL of 2 N HCl, heated at 90 °C for 2 h and then partitioned between ethyl acetate and water. A glycon formononetin was recovered from the ethyl acetate layer and identified by direct comparison with an authentic sample. The water layers were identified by comparison with an authentic sample using TLC analysis^38^. Sugars liberated from compound **6** were identified as glucose.

### Total phenolic content

The total phenolic content (TPC) was determined using a modified version of the Folin-Ciocalteau colorimetric method^39^. The phenolic content was calculated as a gallic acid equivalent from the calibration curve of gallic acid standard solutions (20–300 μg/mL) and was expressed as millimoles of gallic acid equivalent per 100 g of dry weight (mmol equiv. GAE/100 g). The data are reported as the mean ± SD for three replicates.

### Total flavonoid content

The total flavonoid content (TFC) was determined using a sodium borohydride/chloranil-based (SBC) assay^40^. A calibration curve was constructed to create a standard using different concentrations of quercetin (0.1–10.0 mM). The total flavonoid content was expressed as millimoles of quercetin equivalents per 100 g of dry weight (mmol equiv. QUE/100 g). The data are reported as the mean ± SD for three replicates.

### DPPH radical-scavenging assay

The radical scavenging activity of extracts and compounds were measured by a slightly modified version of the method described by Brand-Williams^41^. All the extracts and compound samples were tested in triplicate. The inhibition rate (I%) of the radical-scavenging capacity was calculated using the following equation:


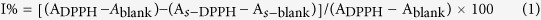


where *A*_DPPH_ is the absorbance of the DPPH solution, *A*_blank_ is the absorbance of methanol instead of DPPH, *A*_s-DPPH_ is the absorbance of the DPPH solution in the presence of the sample, and *A*_s-blank_ is the absorbance of methanol in the presence of the sample.

### ABTS^•+^ radical cation scavenging assay

The ABTS assay was performed as previously described in published literature with some modifications^42^. The results are expressed in terms of micromoles of trolox equivalents per g of dry weight of extracts (μmol eq. trolox/g) or millimoles of trolox equivalent per gram of dry weight of compounds (mmol eq. trolox/g). All determinations were carried out in triplicate.

### Ferric reducing power (FRAP) assay

The FRAP assay used was a modified version of that reported by Benzie and Strain^43^. The FRAP results are expressed in terms of micromoles of trolox equivalents per g of dry weight of extracts (μmol eq. trolox/g) or millimoles of trolox equivalent per gram of dry weight of compounds (mmol eq. trolox/g). All experiments were carried out in triplicate.

### Inhibition of lipid peroxidation

In this assay, a modified thiobarbituric acid reactive species (TBARS) assay was used to measure the amount of lipid peroxide formed. The egg yolk homogenates were used as a lipid-rich medium^44^,^45^. Percentage inhibition was calculated with the following formula:


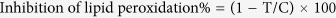


where C is the absorbance of fully peroxidized control and T is the absorbance of test sample. BHA and TBHQ were used as reference standards. The IC_50_ value was calculated from the regression equation obtained from plotting the sample concentration and rate of inhibition.

### Protective effect on H_2_O_2_-induced *E. coli*

The microbial test system used in the manuscript was an effective method to evaluate the antioxidant properties of medicinal plant extracts and polyphenols. This method is easier in terms of experimental operation, lower in cost compared to cellular antioxidant activity assays and more biological relevant than the popular chemistry antioxidant activity measures. In this study, *E. coli* (ATCC No. 25922) was used to determine the antioxidant activity of compounds *in vivo* based on a modified version of the method described by Smirnova^46^,^47^. Bacteria were incubated in Luria-Bertani medium at 37 °C in a 150-mL flask with shaking at 50 r/min overnight until they reached the mid-log phase. The optical density at 600 nm was used to monitor the cell growth. A 1.5-mL cell suspension was added to 8.5 mL of LB medium and resulted in a final OD_600_ of 0.25 ± 0.004. Then, 100 μL of the compounds (0.1 mM), a 1-mL cell suspension and 8.9 mL of LB medium were mixed and incubated at 37 °C with shaking at 180 r/min for 1.5 h. After the 1.5 h incubation, 6.5 mM of hydrogen peroxide was added and the absorbance at 600 nm was then measured immediately. The sample was then incubated at 37 °C with shaking at 180 r/min for an additional 30 min before the absorbance was measured again. The OD_600_ is used to indicate the growth of *E. coli*, with a higher value of the OD_600_ indicating a higher growth rate of *E. coli*. The isolated compounds have the ability to reduce and eliminate the oxidation of H_2_O_2_ and protect the growth of *E. coli*; therefore, the higher antioxidant ability of the compounds indicates that the specific growth rate will be higher.

Specific growth rate was calculated according to the following equation:


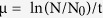


where μ is the specific growth rate and N_0_ and N are the optical density at time zero and t, respectively.

### Statistical analysis

All results are expressed as the mean ± standard deviation (SD). SPSS (Statistical Package for the Social Sciences) software was used to determine significant differences via one-way ANOVA followed by Duncan’s test; values < 0.05 were considered to be significant.

## Additional Information

**How to cite this article**: Pu, W. *et al.* Structural Characterization and Evaluation of the Antioxidant Activity of Phenolic Compounds from *Astragalus taipaishanensis* and Their Structure-Activity Relationship. *Sci. Rep.* **5**, 13914; doi: 10.1038/srep13914 (2015).

## Figures and Tables

**Table 1:** The TPC, TFC and *in vitro* antioxidant activities of extracts and isolated fractions.

Samples	TPC (mmol equiv. GAE/100 g)	TFC (mmol equiv. QUE/100 g)	ABTS (μmol trolox/g)	FRAP (μmol trolox/g)	DPPH (IC_50_ mg/mL)	Lipid peroxidation IC_50_ (mg/mL)
EE	15.20 ± 0.30	110.02 ± 0.65	843.14 ± 4.58^a^	355.85 ± 2.23^a^	0.22 ± 0.003^a^	3.05 ± 0.017^a^
PEF	15.28 ± 0.17	133.30 ± 0.52	823.09 ± 4.65^a^	244.46 ± 2.49^b^	0.19 ± 0.002^b^	2.76 ± 0.025^b^
EAF	46.41 ± 0.34	185.45 ± 1.04	994.50 ± 4.21^b^	685.67 ± 3.21^c^	0.059 ± 0.002^c^	1.95 ± 0.009^c^
BF	23.12 ± 0.62	84.16 ± 0.65	914.31 ± 5.78^c^	395.02 ± 2.62^d^	0.096 ± 0.003^d^	2.27 ± 0.024^d^
WF	5.43 ± 0.23	27.46 ± 0.54	333.93 ± 2.43^d^	126.22 ± 1.98^e^	0.99 ± 0.004^e^	6.48 ± 0.039^e^

**Note:** mean ± SD, N = 3.

The mean values followed by the same small letter did not share significant differences at *p* < 0.05 (Duncan test).

**Table 2:** ^1^H NMR spectroscopic data (chemical shifts [ppm] and coupling constants [Hz]) of isolated compounds (500 MHz, DMSO-d_6_).

Position	Compounds					
	1	2	3	4	5	6
1						
2	4.21 d (10.3, H-2b) 3.94 t(H-2a)	8.34 s	7.65 d (1.2)			8.45 s
3			6.87 d (8.8 )			
4	2.92 (H-4b), 2.78 (H-4a)					
5	6.85 d (8.4)	7.98 d (8.5)	6.87 d (8.8)			8.05 d (10.0)
6	6.22 s	6.95 dd (2.0, 2.0)	7.65 d (1.2)	6.19 d (2.0)	6.19 d (2.0)	7.16 d (2.0)
7						
8	6.30 d (8.0)	6.88 d (2.5)		6.42 d (2.0)	6.45 d (2.0)	7.25 s
9						
10						
1′						
2′		7.52 d (8.5)		7.65 d (8.5)	8.04 d (8.5)	7.54 d (2.0)
3′		7.00 d (9.0)	6.30 d (2.4)		6.92 d (8.5)	7.00 d (2.0)
4′						
3′O-CH3	3.78 s (3H)					
4′O-CH3	3.79 s (3H)	3.80(3H,s)				3.80 s (3H)
5′	6.44 d (8.4)	7.00 d (9.0)	6.43 dd (2.4, 2.4)	6.89 d (6.8)	6.92 d (8.5)	7.00 d (2.0)
6′	6.75 d (8.8)	7.52 d (8.5)	7.99 d (8.8)	7.54 dd (2.0, 2.0)	8.04 d (8.5)	7.54 d (2.0)
1″						5.16 d (4.8)
2″						
3″						
4″						
5″						
6″						
α			7.63 d (5.2)			
β			7.81 d (15.2)			
CO						

**Table 3:** 13C NMR spectroscopic data (chemical shifts [ppm]) of isolated compounds (500 MHz, DMSO-d_6_).

Position	Compounds					
	1	2	3	4	5	6
1			126.44			
2	73.51	153.60	130.43	147.28	147.29	154.15
3	36.08	123.63	115.54	136.14	136.10	123.84
4	33.84	175.08	160.22	176.27	176.35	175.15
5	133.75	127.76	115.54	161.13	161.15	127.44
6	111.59	115.67	130.43	98.65	98.67	116.11
7	160.09	163.05		164.30	164.35	161.91
8	106.89	102.59		93.85	93.96	103.86
9	158.92	157.92		156.60	156.64	157.52
10	117.35	117.08		103.45	103.49	118.91
1′	124.98	124.71	113.15	120.51	122.12	124.47
2′	152.08	130.54	165.54	148.13	129.97	130.57
3′	140.06	114.08	102.50	145.49	115.91	114.10
4′	155.66	159.43	166.18	115.47	159.63	159.49
3′O-CH3	63.61					
4′O-CH3	58.78	55.62				55.62
5′	125.36	114.08	107.99	116.05	115.91	114.10
6′	106.36	130.54	131.97	122.40	129.97	130.57
1″						100.42
2″						73.59
3″						76.93
4″						70.07
5″						77.67
6″						61.09
α			116.95			
β			144.16			
CO			192.01			

**Table 4:** *In vitro* antioxidant activities of isolated compounds from *A.taipaishanensis.*

Compounds	ABTS mmol trolox/g	FRAP mmol trolox/g	DPPH IC_50_ (μM)	Lipid peroxidation IC_50_(μM)
1	28.62 ± 0.27^a^	10.77 ± 0.11^a^	0.30 ± 0.003^a^	1.87 ± 0.07^a^
2	14.18 ± 0.20^b^	3.27 ± 0.05^b^	1.16 ± 0.009^b^	2.69 ± 0.11^b^
3	18.83 ± 0.18^c^	3.83 ± 0.05^c^	0.83 ± 0.007^c^	2.29 ± 0.13^c^
4	34.66 ± 0.20^d^	14.18 ± 0.13^d^	0.09 ± 0.001^d^	1.65 ± 0.08^d^
5	31.79 ± 0.13^e^	14.13 ± 0.16^d^	0.14 ± 0.002^d^	1.30 ± 0.09^e^
6	12.67 ± 0.16^f^	1.10 ± 0.03^e^	1.52 ± 0.06^e^	2.73 ± 0.11^b^
BHA	4.24 ± 0.10^g^	5.83 ± 0.08^f^	7.54 ± 0.38^f^	0.68 ± 0.05^f^
TBHQ	3.36 ± 0.08^h^	6.57 ± 0.13^g^	3.66 ± 0.16^g^	0.69 ± 0.03^f^

**Note:** mean ± SD, N = 3.

The mean values followed by the same subscript letter did not share significant differences at p < 0.05 (Duncan test).

Compound 1: 7, 2′-dihydroxy-3′, 4′-dimethoxy-isoflavane; compound 2: Formononetin; compound 3: Isoliquiritigenin; compound 4: Quercetin; compound 5: Kaempferol; compound 6: Ononin.
